# Rational design of a quantitative, pH-insensitive, nucleic acid based fluorescent chloride reporter†
†Electronic supplementary information (ESI) available. See DOI: 10.1039/c5sc04002g


**DOI:** 10.1039/c5sc04002g

**Published:** 2015-12-01

**Authors:** Ved Prakash, Sonali Saha, Kasturi Chakraborty, Yamuna Krishnan

**Affiliations:** a Department of Chemistry and the Grossman Institute , University of Chicago , 929E, 57th Street, E305A, GCIS , Chicago , Illinois 60637 , USA . Email: yamuna@uchicago.edu; b National Centre for Biological Sciences , TIFR, GKVK, Bellary Road , Bangalore 560065 , India

## Abstract

Chloride plays a major role in cellular homeostasis by regulating the lumenal pH of intracellular organelles. We have described a pH-independent, fluorescent chloride reporter called *Clensor* that has successfully measured resting chloride in organelles of living cells. Here, we describe the rational design of *Clensor*. *Clensor* integrates a chloride sensitive fluorophore called 10,10′-bis[3-carboxypropyl]-9,9′-biacridinium dinitrate (BAC) with the programmability, modularity and targetability available to nucleic acid scaffolds. We show that simple conjugation of BAC to a DNA backbone fails to yield a viable chloride-sensitive reporter. Fluorescence intensity and lifetime investigations on a series of BAC-functionalized structural variants yielded molecular insights that guided the rational design and successful realization of the chloride sensitive fluorescent reporter, *Clensor*. This study provides some general design principles that would aid the realization of diverse ion-sensitive nucleic acid reporters based on the sensing strategy of *Clensor*.

## Abstract 2

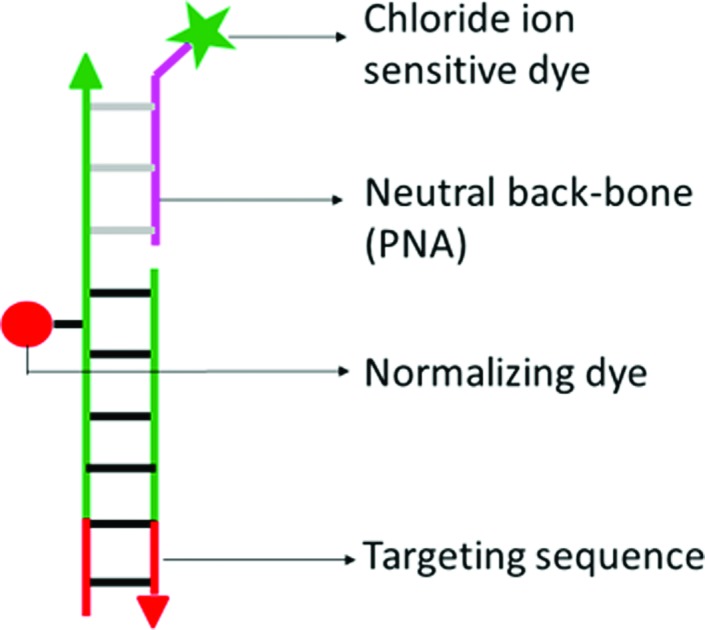
This study describes the rational design of a DNA-based chloride reporter.

## Introduction

Cellular metabolic homeostasis is controlled by a wide range of chemical processes occurring in an orchestrated fashion inside specific sub-cellular organelles. Ions play a major role in defining the chemistry occurring within a specific organelle, thereby determining the fate of molecular cargo trafficking along a given pathway. Lumenal pH is one of the major regulators of these pathways.^1^ For example, the mannose-6-phosphate dependent cargo sorting occurring in the Golgi works on the basis of a pH gradient.^2^ It is well-known that chloride is a primary facilitator of organelle acidification,^3^ and its imbalance leads to diseased conditions like cystic fibrosis and lysosomal storage disorders.^4^,^5^

There are several reports on measurement of cytosolic chloride concentration inside live cells.^6^–^12^ However the ability to accurately measure resting chloride in subcellular organelles has proved challenging due to their diverse resting pH values. As most chloride reporters based on YFP are also pH sensitive,^6^ and further as intracellular chloride affects lumenal pH,^5^ there is a need for a pH insensitive reporter that can measure chloride. Our laboratory has shown that DNA based nanodevices can function as highly sensitive, bright fluorescent reporters inside subcellular organelles of live cells and live organisms.^13^–^16^ We have recently shown that a DNA-based nano-device called *Clensor* functions as a pH-independent, fluorescent, ratiometric chloride ion reporter inside sub-cellular organelles of living cells.^17^

We reasoned that the introduction of a chloride sensitive dye onto a DNA-based scaffold bearing a single normalizing fluorophore would yield a DNA nano-device that could be targeted to a specific organelle. This DNA nano-device would be able to quantitate resting chloride in the target organelle as it would comprise a chloride sensitive fluorophore and a normalizing fluorophore. Given that the nano-device would be comprised of two complementary strands bearing each of these fluorophores, this would result in a reporter system that would be monodisperse in bulk, with a 1 : 1 ratio of the reporter and normalizer fluorophores. Such monodispersity is key to accuracy in quantitation of sub-cellular chloride in living systems.

**Fig. 1:**
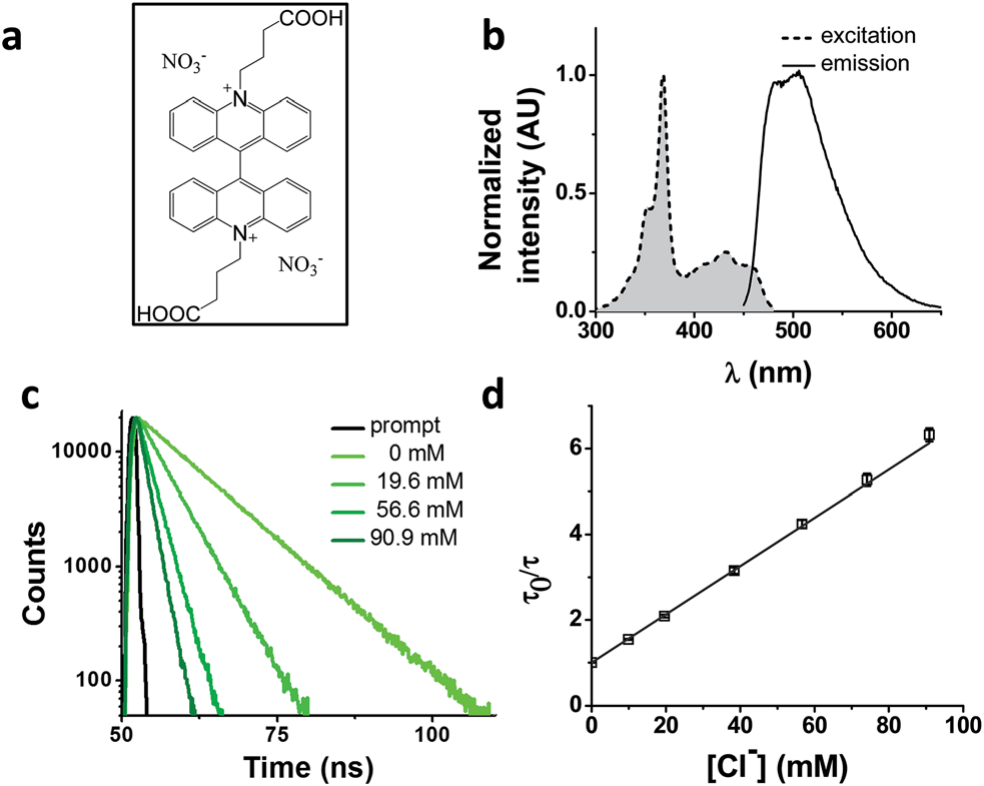
Photophysical properties of 10,10′-bis[3-carboxypropyl]-9,9′-biacridinium dinitrate (BAC). (a) Chemical structure of BAC. (b) Excitation and emission spectra (*λ*_ex_ = 443 nm) of BAC. (c) Representative fluorescence lifetime decay traces of 1 μM BAC in 10 mM potassium phosphate buffer, pH 7.2 at various chloride concentrations (*λ*_ex_ = 443 nm, *λ*_em_ = 488 nm, RT). (d) Stern–Volmer plot for BAC. Error bars indicate the mean of three independent experiments ± s.e.m.

We chose as our chloride reporter BAC (Fig. 1a), a quinolinium-based dye, well-known to undergo collisional quenching in presence of chloride.^6^ Such quinolinium-based chloride indicators have good sensitivity and rapid response (<1 ms) to changes in Cl^–^ concentration.^18^ Even though BAC is sensitive to collisional quenching by all halides,^19^ concentration of other halides (F^–^, Br^–^ and I^–^) and pseudo halides (CN^–^, SCN^–^ and azide) in biological systems combined is less than 1% the value of chloride.^20^–^24^ Hence, in living systems, BAC predominantly behaves like a chloride sensor.^6^ This sensor is designed to act as a chloride sensor only in living systems and not for other applications like environmental remediation. Importantly, they are insensitive to physiological changes in pH. BAC was chosen due to its long excitation and emission wavelengths relative to other quinolinium based reporters.^18^,^19^,^25^ Further, BAC has been used to quantitate chloride in endosomes, albeit with much lower accuracy.^6^

## Results and discussion

### Photophysical characterization of BAC

BAC shows a mono-exponential fluorescence decay in 10 mM potassium phosphate buffer (pH 7.2) with a lifetime of 9.15 ns (Table 1). It shows sensitivity towards chloride with a *K*_SV_ of 56.4 M^–1^ and *k*_q_ of 6.16 ns^–1^ M^–1^. *K*_SV_ values obtained from fluorescence quenching experiments reveal overall sensitivity of fluorophore to quencher, while the *k*_q_ values give information about rate of diffusion of quencher around sensing dye. The lower value of *k*_q_ compared to diffusion limited rate (10 ns^–1^ M^–1^) is likely due to the two negatively charged carboxyl groups shielding the positively charged core of the dye, thereby reducing the efficiency of collisional quenching by negatively charged chloride ions.^26^ In fact, it has been reported that synthetic analogues of BAC show different values of *k*_q_ depending upon the nature of the charge on the side chain.^19^

**Table 1:** Fluorescence lifetimes (*τ*_0_), their relative populations (shown in brackets), average lifetime (*τ*_0 Avg_), Stern–Volmer constant (*K*_SV_), bimolecular quenching rate constant (*k*_q_) and fold change observed for various versions of nucleic acid based chloride sensors. Insensitive component is shown in italics. *Fold change in *τ*_Avg_, **fold change in fluorescence intensity from 24.4–90.9 mM [Cl^–^]

Construct	*τ*_0_ (ns)	*τ*_0 Avg_ (ns)	*K*_SV_ (M^–1^)	*k*_q_ (M^–1^ ns^–1^)	Fold change*	Fold change**	*χ*_red_^2^
BAC	9.15 ± 0.01	9.15 ± 0.01	56.4 ± 0.6	6.16 ± 0.07	2.60	2.89	1.00
BAC–ssDNA	5.47 ± 0.25 (98 ± 1), *13.94 ± 0.42 (2 ± 1)*	5.47 ± 0.25	33.56 ± 0.76	6.13 ± 0.41	2.22	1.24	1.03
BAC–dsDNA_B0_	1.27 ± 0.2 (38 ± 11), 5.78 ± 0.08 (60 ± 10), *13.00 ± 0.13 (2 ± 1)*	5.16 ± 0.19	33.6 ± 1.78	6.52 ± 0.59	2.27	1.21	0.98
BAC–dsDNA_B1_	1.1 ± 0.08 (74 ± 0), 4.43 ± 0.21 (16 ± 0), *13.5 (9 ± 1)*	2.68 ± 0.11	3.9 ± 0.5	1.45 ± 0.45	1.3	1.3	1.04
BAC–dsDNA_B2_	1.1 ± 0.08 (44 ± 10), 4.44 ± 0.04 (35 ± 7), *13.5 (22 ± 2)*	3.57 ± 0.26	7.7 ± 1.1	2.15 ± 0.23	1.3	1.3	0.98
BAC–dsDNA_B3_	1.29 ± 0.1 (85 ± 2), 7.79 ± 0.19 (11 ± 1), *14.51 ± 0.12 (3 ± 1)*	4.17 ± 0.11	ND	ND	ND	1.27	0.97
BAC + free dsDNA	9.11 ± 0.5 (69.6%), 5.72 ± 0.76 (29.4%), *13.5 ± 0.45 (1)*	8.4	56.1	6.68	NA	NA	0.94
BAC–ssPNA	4.34 ± 0.17 (41 ± 2), 8.44 ± 0.11 (59 ± 2)	7.35 ± 0.06	64.3 ± 1.1	8.74 ± 0.22	2.68	1.82	0.98
BAC–dsPNA	5.02 ± 0.14 (47 ± 3), 9.17 ± 0.17 (53 ± 3)	7.80 ± 0.08	70.8 ± 0.2	9.02 ± 0.11	2.72	2.72	1.01
*Clensor*	3.9 ± 0.03 (35 ± 1), 8.3 ± 0.01 (65 ± 1)	7.40 ± 0.01	58.6 ± 0.3	7.92 ± 0.05	2.61	1.82	0.97

### Effect of conjugation to DNA

Previously, it has been shown that due to the presence of the negatively charged phosphate backbone, DNA nanodevices act as anionic ligands and are internalized by *Drosophila* hemocytes that express the ALBRs (Anionic Ligand Binding Receptors).^13^,^15^ Our laboratory has also demonstrated a general strategy to target DNA nanostructures, carrying a specific dsDNA sequence d(AT)_4_ to probe the ionic environment of the lumen of endocytic organelles.^14^

**Fig. 2:**
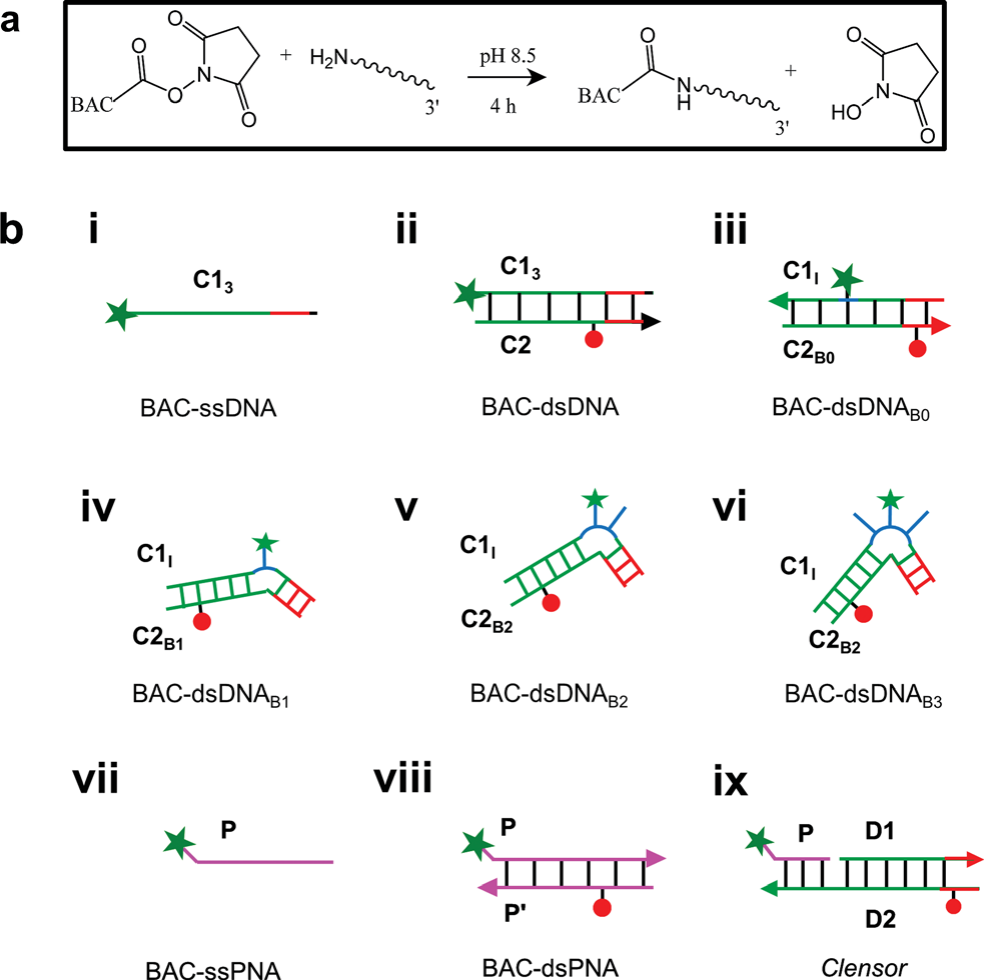
(a) Reaction scheme for chemical conjugation of BAC to amine labeled DNA. (b) Schematic showing structures of different chloride sensing nucleic acids constructs. BAC: green filled star; Alexa 647: red filled circle.

In order to achieve targeted intracellular chloride sensing in a specific organelle of interest, we conjugated BAC to single stranded DNA at the 3′ end *via* a flexible hexamethylene linker using hydroxysuccinimide chemistry (BAC–ssDNA, Fig. 2a). Upon conjugation, the fluorescence intensity of the dye as well as its sensitivity towards chloride reduced (Table 1).

This reduction in fluorescence intensity as well as lifetime upon conjugation to DNA is due to Photoinduced Electron Transfer (PET) from guanine to fluorophore^27^–^29^ that is dependent upon the relative redox potentials of the dye and nucleobase.^29^ The energetics and feasibility of this electron transfer process, described by the Weller's equation^30^ indicates that guanine is mainly responsible for PET based quenching by nucleobases.^29^ The efficiency of PET from guanine reduces logarithmically with increasing distance.^30^

We observed a similar trend in fluorescence lifetime where we observed bi-exponential decay with lifetime values of 5.5 ns (98%) and 13.9 (2%) (Table 1 and S3 in the ESI†). BAC–ssDNA incorporates 18% guanine residues where the first guanine is located 10 nt apart from the BAC labeled 3′ end (Table S2 in the ESI†). Given that the persistence length of ssDNA is only 3 bases,^31^ potentially BAC–ssDNA can collapse into a globular conformation bringing the guanine residues significantly nearer to BAC. Therefore, we assigned the 5.5 ns component to this quenched state of BAC that has 98% relative contribution to the total fluorescence intensity (Table 1). The reduction in the lifetime value of the major component accounts for the reduction of fluorescence intensity.

This pre-quenched state of BAC with 5.5 ns lifetime exhibited 40% reduction in chloride sensitivity showing a *K*_SV_ value of 33.56 M^–1^ compared to free BAC (*K*_SV_ = 56.4 M^–1^) (Table 1). The *k*_q_ value remained unaffected upon conjugation to DNA indicating that the negatively charged DNA backbone provides similar level of shielding as that of two carboxylate groups, and hence negligible change in chloride diffusion around BAC–ssDNA.

**Fig. 3:**
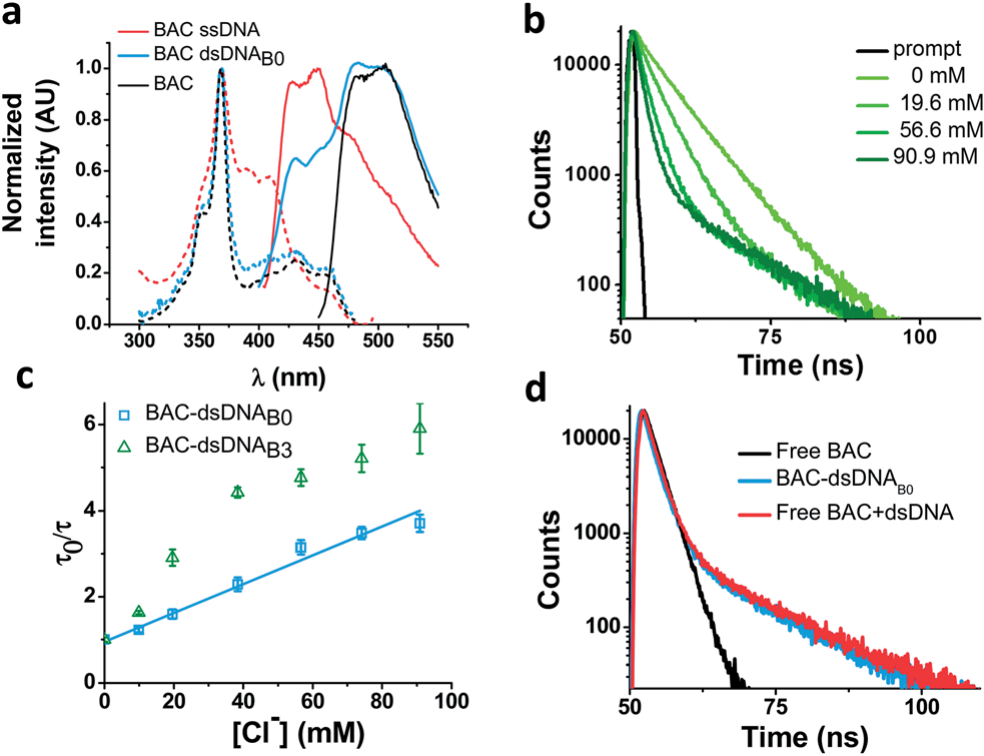
Interaction of BAC with DNA (a) comparison of excitation and emission spectra of free BAC, BAC–ssDNA and BAC–dsDNA_B0_. (b) Representative fluorescence lifetime decay traces of 1 μM BAC–dsDNA_B0_ in 10 mM potassium phosphate buffer, pH 7.2 at various chloride concentrations (*λ*_ex_ = 443 nm, *λ*_em_ = 488 nm). (c) Stern–Volmer plots for BAC–dsDNA_B0_ and BAC–dsDNA_B3_. Average lifetime (*τ*_Avg_) of the sensitive components has been used to plot Stern–Volmer plot. Error bars indicate the mean of two independent experiments ± s.e.m. (d) Representative fluorescence lifetime decay traces of 1 μM BAC–dsDNA_B0_, BAC and 2 μM BAC + 1.5 mM_b_ dsDNA in 10 mM potassium phosphate buffer (pH 7.2) at 60 mM chloride (*λ*_ex_ = 392 nm, *λ*_em_ = 488 nm).

In order to understand the nature of the longer lifetime component observed for BAC–ssDNA, we determined the fluorescence lifetime of free BAC in the presence and absence of excess dsDNA (Fig. 3d and Table 1). When dsDNA was added to a solution containing free BAC, the observed fluorescence decay (Table 1) yielded a tri-exponential decay model as the best fit to the data with decay components of 5.7, 9.1 and 13.5 ns. The 13.5 ns component proved to be insensitive to chloride quenching, indicating that this lifetime originates due to association of BAC with dsDNA. Spectroscopic studies have shown that lucigenin, the parent molecule of BAC, intercalates efficiently into the DNA double helix.^32^ Electrostatic interaction between cationic lucigenin and the polyanionic DNA play a crucial role in the propensity of lucigenin to intercalate in dsDNA.^32^ It is well known that intercalated lucigenin is also less sensitive to quenching by chloride. Free BAC is also expected to intercalate into dsDNA analogous to its parent molecule lucigenin and show an increased lifetime because of hindered movement. In fact, fluorescence lifetimes of other fluorophores like ethidium bromide and acridine orange increase to 1400% and 300% respectively upon binding dsDNA,^27^ and are also considerably less sensitive to quenching due to inaccessibility.^33^ However, the bound form of BAC with 13.5 ns lifetime represents only 1% population in contrast to 98% for ethidium bromide and 88% for acridine orange indicating a weaker interaction.^27^

Closer scrutiny of the excitation spectra of BAC–ssDNA and BAC–dsDNA, revealed that intensity of 443 nm band was reduced, while that of 392 nm was increased (Fig. 3a). We observed similar trends in the emission spectrum of BAC–dsDNA, where we observe a blue shifted emission at 425 nm along with the normal emission (Fig. 3a), that is contrary to most cases where intercalation or groove binding of a dye leads to a small red shift in its absorption spectrum.^33^ The nature of molecular interaction responsible for this is as yet unclear, but it is worth mentioning that blue shifts usually occur when the dye interacts with single stranded oligonucleotide^34^ or because of reduced solvation.^35^,^36^ In order to check whether BAC is solvent accessible, we titrated BAC–ssDNA against chloride and observed a significant population (27%) that was insensitive to quenching at 392 nm excitation (Fig. S2d in the ESI†), indicating that it is indeed solvent inaccessible. On the other hand, almost the entire population (98%) is sensitive to chloride at 443 nm excitation (Table 1). This indicates that 392 nm excites the insensitive form which has a blue shifted excitation with respect to the free dye. Notably, the reduction in sensitivity due to DNA binding is negligible when excited at 443 nm because the emission arising from it is blue shifted (Fig. S2c in the ESI†), and hence, has a meagre contribution towards total population (2%). But as we have observed using 392 nm excitation, a significant population is insensitive to quenching because of ssDNA binding, which is an indicator of an inefficient sensor.

### Effect of DNA structure

The ability of guanine to quench fluorophores strongly depends on its hydrogen bonding state and its position within the double-stranded structure.^37^ It has also been shown that depending on sequence, a fluorescein labeled oligo undergoes de-quenching upon hybridization. Duplexes containing dA–T base pairs adjacent to covalently labeled fluorophores show 14% higher fluorescence intensities compared to their single stranded forms.^37^ Therefore, to study the effect of duplexation on the chloride sensitivity of BAC, BAC–ssDNA was hybridized to oligo C2 to form BAC–dsDNA and fluorescence measurements were carried out. Upon comparison of the excitation and emission spectra of BAC–dsDNA with that of BAC–ssDNA, we observed that intensity of 443 nm band had recovered, while that of 392 nm had reduced (Fig. 3a) indicating a reduction in the DNA-complexed–likely intercalated–form of BAC. We expected BAC–dsDNA to show higher fold change in R/G or higher chloride sensitivity than BAC–ssDNA due to relief of quenching as the 3′ end of BAC–ssDNA is highly rich in AT. In contrast, BAC–dsDNA showed negligible increase in chloride sensitivity (Table 1). Thus, based on fluorescence intensity and lifetime data, we may conclude that the reduced chloride sensitivity of BAC in BAC–ssDNA is primarily due the prior quenching of BAC by guanine residues.^28^,^37^ Additionally, the complexation or intercalation of BAC into either dsDNA or any secondary structure formed by ssDNA might also restrict the accessibility of BAC to chloride thereby decreasing the chloride sensitivity.

Next, we sought to understand whether there was any positional effect of BAC attachment on the DNA scaffold or effect of linker flexibility on chloride sensitivity of BAC. Therefore, we chose oligo C1_I_, where BAC is internally conjugated to T_24_ using a rigid 5-aminoallyl linker. The internally labeled oligo C1_I_ was hybridized to C2_B0_ to form BAC–dsDNA_B0_ (Fig. 2biii). Moreover to minimize groove binding of BAC into nearby base pairs as well as to understand the effect of flanking of base pairs on chloride sensitivity of BAC, we designed bulges of different sizes around the point of BAC attachment (T_24_) by changing the complementary sequence as shown in Fig. S3 in the ESI.† In case of BAC–dsDNA_B1_, an unopposed T_24_ residue, flanked by C residues is known to remain extrahelical^38^ and hence is expected to position BAC away from helical region. However, the conformation adopted by multiple pyrimidines comprising the bulge for BAC–dsDNA_B2_ (dCdT) and BAC–dsDNA_B3_ (dCdTdC) is not described^39^ although increase in bulge size is known to enhance the conformational flexibility of the bulged nucleotides.^40^

We therefore determined the chloride sensitivity of BAC when incorporated internally into the above designed bulges in DNA assemblies using fluorescence intensity based chloride titrations (Table 1). All the internally labeled constructs showed similar fold changes in R/G (1.2–1.5) with their single stranded forms indicating no significant effect of bulge positioning using a rigid linker on the chloride sensitivity of BAC. To investigate the chemical environment of BAC in these bulge-labeled scaffolds, we determined fluorescence lifetimes in the absence (BAC–dsDNA_B0_) and presence of a pyrimidine-rich (BAC–dsDNA_B3_) bulge around the BAC label (T_24_). BAC–dsDNA_B0_ showed complex multi-exponential fluorescence decay with components of 1.3, 5.8 and 13 ns (Table 1). We can assign the 13 ns lifetime component to the intercalated or complexed state of BAC, while 1.3 ns and 5.8 ns as quenched states as seen from BAC–ssDNA (Table 1). However the relative contributions of these forms are now altered as we go from BAC–ssDNA to BAC–dsDNA_B0_. Here, the 1.3 ns component corresponds to a population (38%) of BAC that is more efficiently quenched by the three guanine residues within 4 nt on either side of the dye. In fact, for a dye like fluorescein, positioning the fluorophore internally leads to far more efficient quenching than when positioned at the 5′ or 3′ terminus.^37^

Both, BAC–dsDNA_B1_ as well as BAC–dsDNA_B2_ showed complex multi-exponential fluorescence decay (Table 1). We observed considerable reduction in fluorescence lifetime, *K*_SV_ and *k*_q_ compared to BAC–dsDNA_B0_. With the introduction of a bulge, the accessibility of chloride to BAC is reduced as much as 3–4 fold.^41^ BAC–dsDNA_B2_ and BAC–dsDNA_B3_ shows only an ∼33% and 54% improvement respectively in fluorescence lifetime compared to that of BAC–dsDNA_B1_ indicating that increasing the bulge size does little to improve the sensitivity of BAC to chloride.

BAC–dsDNA_B3_ also showed similar complex multi-exponential fluorescence decay with components of 1.3, 7.8 and 14.5 ns (Table 1). It is known that bulge structures significantly affect the local geometry of DNA and RNA duplexes.^42^ Diverse biophysical experiments such as electrophoretic mobility,^43^,^44^ FRET,^45^ electron microscopy^46^ and NMR^39^ suggest that bulges introduce a defined kink into the helical axis of the DNA and RNA molecules. The magnitude of helix bending depends on the size and the sequence of the bulge.^47^,^48^ Purine bulges generate greater helical bending than pyrimidine bases.^47^ FRET efficiency are very similar for both DNA and RNA helices containing bulges, indicating a similar kinking process despite the geometrical differences between DNA and RNA helices.^45^ The estimated bend angle for UUU bulge is 55°.^48^ Molecular modelling based on NMR data indicated that the angle of bending at the bulge site ranges between 50° and 60° in the direction away from the bulge containing strand for an ATA bulge.^39^ Further a significant reduction in helical twist was also observed due to insertion of three nucleotide bulge.^39^

We considered a 55° bend angle for BAC–dsDNA_B3_ to calculate relative change in distance between BAC and the nearby guanine residues upon bulge insertion. To calculate the minimum distance we did not incorporate any helical twist in the calculation. In this case, the distance between BAC and the nearest guanine residue (one nucleotide away from T_24_) on the 3′ increases from 15.2 Å to 19.9 Å (Fig. S3 in the ESI†). Similarly the distance between BAC and the nearest guanine residue (three nucleotides away from T_24_) on the 5′ side increases from 19.2 Å to 26.6 Å. The new component with 7.8 ns lifetime likely reflects the reduced quenching efficiency by guanine because of the greater distance between BAC and its neighboring guanines due to bending caused by introduction of the bulge.^28^ However, due to the small percentage of this population (11%), the overall chloride sensitivity of BAC remains poor. The major population (85%) has a short lifetime of 1.3 ns indicating a conformer with highly efficient quenching by guanine present at the edge of the bulge. This implies that for the majority of time, BAC in this conformer prefers accommodation within the bulge, where it interacts strongly with the nucleobases and the backbone. Fluorescence lifetime based chloride titration of BAC–dsDNA_B0_ and BAC–dsDNA_B3_ revealed that the 14.5 ns lifetime species was again present as the chloride insensitive bound/intercalated form. Lastly, the intrinsic structural dynamics of the dsDNA backbone yields non-linearity in the Stern Volmer plot of BAC–dsDNA_B3_ (Fig. 3c), for chloride concentrations >40 mM and this portion was not considered for analysis.

Cumulatively, DNA assemblies carrying an internal BAC label exhibit two types of quenched states depending on the quenching efficiency of the guanine residues (Table 1). The efficiency of quenching by guanine residues and relative contributions of both quenched states depends on the geometry around the internal fluorophore label. Importantly, a chloride insensitive 14 ns component was constantly observed in all three dsDNA constructs independent of fluorophore position, linker flexibility and nature of base pairs in the proximity, which correlates well with a form of the fluorophore that is bound/intercalated with DNA. Importantly, we do not observe any component with lifetime comparable to that of free dye, indicating a strong interaction between DNA and BAC; observed as (i) PET based quenching and (ii) dsDNA intercalation.

### Effect of an electrically neutral backbone

An ideal chloride reporter should have a high fluorescence intensity as well as high chloride sensitivity. It is known that quenching efficiency of I^–^ decreases with increase in negative charge on the fluorophore due to the repulsion between the quencher and the negative charge on the fluorophore.^26^ Increase in ionic strength compensates this by shielding the negative charge on the fluorophore. Accordingly, conjugation of BAC to DNA not only reduces its fluorescence intensity, but also its sensitivity towards collisional quenching by chloride. Thus, we hypothesized that the negatively charged DNA backbone attracts the positively charged quinolinium core of BAC and this proximity to DNA enhances quenching of BAC by nucleobases resulting in reduced chloride sensitivity.^33^,^35^,^49^

Electrostatic interactions are a primary contributor to fluorophore–DNA interactions.^50^ We reasoned that eliminating these electrostatic interactions, would yield an assembly with higher chloride sensitivity. We therefore covalently linked BAC to a PNA oligomer because it has a neutral backbone (BAC–ssPNA). Peptide nucleic acids are remarkable DNA/RNA mimics in which the sugar–phosphate backbone of the DNA/RNA is replaced by a synthetic peptide backbone formed from *N*-(2-aminoethyl)-glycine units.^51^,^52^ The plot of R/G *vs.* [Cl^–^] showed 1.8 fold change in R/G compared to 1.2 observed for BAC–ssDNA (Table 1) indicating an increased chloride sensitivity of BAC upon conjugation to PNA. Next to study the effect of hybridization, BAC–ssPNA was hybridized to P′ to form BAC–dsPNA (Fig. 2bviii). Upon hybridization, the fold change in R/G values increased to 2.7 which is indeed comparable to fold change observed for free BAC.

Fluorescence lifetime experiments were carried out to ascertain the mechanism of improvement of chloride sensitivity upon conjugation to PNA. BAC–ssPNA showed a biexponential fluorescence decay with components of 4.3 and 8.4 ns (Table 1). The sequence of P contains 8% guanine residues (Table S2 in the ESI†). Neutral polymers are known to collapse into dense spherical globules in order to reduce unfavorable solvent polymer interactions and charged polymers show a higher radius of gyration.^53^ Further given its neutral backbone and much higher conformational flexibility of BAC–ssPNA, compared to ssDNA the average proximity of guanine is likely to be much shorter in random coiled BAC–ssPNA. Therefore, we assign 4.3 ns lifetime component as the guanine quenched population of BAC due to PET. However, 59% of BAC remained unaffected resulting in a 8.4 ns lifetime that is comparable to free BAC (9.2 ns), indicating weakened interaction between PNA and BAC compared to DNA and BAC. BAC–dsPNA also showed two components with 5 ns and 9.2 ns lifetimes (Table 1) indicating that a major population of BAC is in same environment as free BAC. This reinforces that there is some quenching of BAC due to the DNA scaffold itself, before the addition of chloride, which is responsible for the lower chloride sensitivity in BAC–ssDNA and BAC–dsDNA.

Interestingly, BAC–ssPNA and BAC–dsPNA did not show the 14 ns lifetime component. It is known that the propensity of EtBr to intercalate reduces drastically as DNA–DNA > DNA–PNA > PNA–PNA.^50^ Fluorescence lifetime based chloride titrations of BAC–ssPNA and BAC–dsPNA showed *K*_SV_ values slightly better than that of the free BAC (Table 1). The 42% and 47% increase in observed *k*_q_ value for BAC–ssPNA and BAC–dsPNA respectively indicate higher diffusion rate of chloride in this medium than that of BAC–ssDNA. This is consistent with the negative charge due to DNA surrounding BAC in BAC–ssDNA restricts chloride diffusion rate, and is partially responsible for reduction in chloride sensitivity of DNA-based assemblies.

### *Clensor* 

To combine the dsDNA specific intracellular targeting and increased chloride sensitivity based on our previous observation, we designed a scaffold consisting of three nucleic acids oligomers, that we call *Clensor*. In *Clensor*, BAC has been covalently conjugated to the N-terminus of ssPNA using hydroxysuccinimide chemistry to give BAC ssPNA. BAC ssPNA has then been hybridized to its complementary DNA stand to form a DNA–PNA hybrid duplex. PNA oligonucleotides containing purine and pyrimidine nucleobases hybridize with complementary DNA and RNA strands to form right-handed, double-helical complexes according to the Watson–Crick rules of hydrogen bond mediated base pair formation.^54^,^55^ The hybrid duplexes formed by PNA with DNA generally have higher thermal stabilities than their duplex DNA counterparts.^56^ They show unique ionic strength effects because the PNA strand does not bear negatively charged phosphate groups. Examination of the PNA/DNA hybrid structure by NMR spectroscopy reveals a unique double helical conformation that has features of both A and B-form DNA.^55^

*Clensor* is composed of three modules: a sensing module (P), a normalizing module (D2) and a targeting module (D1) (Fig. 2bix and 4b). The sensing module, P is a 12 mer peptide nucleic acid (PNA) oligomeric sequence conjugated to BAC. The normalizing module, D2 is a 38 nt DNA sequence carrying an Alexa 647 fluorescent label that is Cl^–^ insensitive. The targeting module, D1, is a 26 mer DNA sequence. P and D1 are hybridized to adjacent sites on D2 as shown in Fig. 2bix and 4b. The dsDNA domain on *Clensor* comprising D1 and D2 functions as a negatively charged ligand for the ALBRs (Anionic Ligand Binding Receptors)^16^ and harbors the d(AT)_4_ sequence required for targeting of the sensor.^14^

**Fig. 4:**
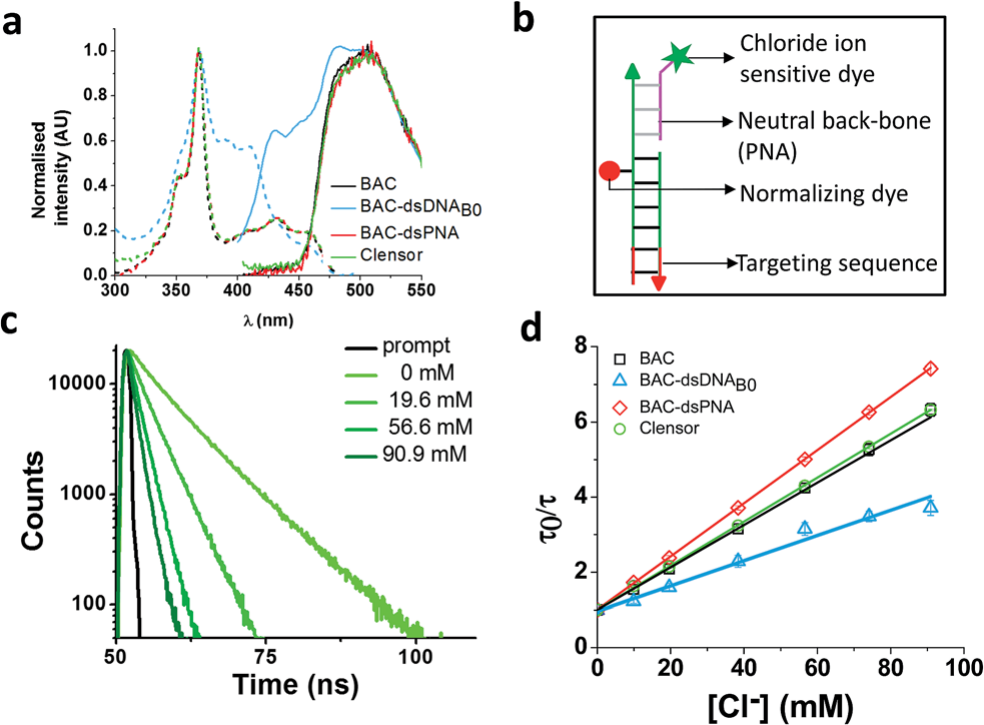
(a) Comparison of excitation and emission spectra of BAC, BAC–dsDNA_B0_, BAC–dsPNA and *Clensor*. (b) Schematic of *Clensor*. (c) Representative fluorescence lifetime decay traces of 1 μM *Clensor* in 10 mM potassium phosphate buffer, pH 7.2 at various chloride concentrations (*λ*_ex_ = 443 nm, *λ*_em_ = 488 nm). (d) Stern–Volmer plots for BAC, BAC–dsDNA_B0_, BAC–dsPNA and *Clensor*. Average lifetime has been used to evaluate *K*_SV_ and *k*_q_. Error bars indicate the mean of three independent experiments ± s.e.m.

*Clensor* showed 1.8 fold change in R/G in the physiological range of chloride concentration when subjected to fluorescence quenching by addition of NaCl (Table 1). It showed 33% improvement in R/G fold change compared to BAC–dsDNA_B0_. However 33% decrease in R/G fold change of *Clensor* compared to BAC–dsPNA can be explained by the presence of the negatively charged DNA backbone in the hybrid. Fluorescence lifetime experiments on *Clensor* revealed two components with 4.0 ns and 8.3 ns lifetime, as observed as BAC–ssPNA (Table 1). However, their relative contributions are different from BAC–ssPNA. The increase in relative contribution of the 8.4 ns component with from 59% to 65% is probably due to the relief of quenching effect upon hybridization. Again, no bound/intercalated population of BAC corresponding to 14 ns was observed in *Clensor* as expected. Fluorescence lifetime based chloride titration revealed a *K*_SV_ value of 58.6 M^–1^ that is comparable with free BAC indicating a similar sensitivity of BAC to quencher (Table 1). However, the 29% increase in *k*_q_ value indicates that negatively charged DNA backbone of the hybrid duplex is responsible for the lower diffusion rate of chloride. Table 1 summarizes the photophysical properties and sensitivities of various constructs. However, there is a slight mismatch between the intensity fold change and lifetime fold change possibly because insensitive component has been excluded from the calculation for lifetime fold change; but overall trend seems to be conserved.

## Conclusions

The goal of this study was to merge the reporter capabilities of an organic dye like BAC with the programmability of the DNA scaffold. Using various photophysical studies, we have described the rational design of a nucleic acid scaffold that successfully integrates the chloride sensing ability of an organic dye. This turns out to be non-trivial, as the functionality of such ion-based fluorescent reporters becomes highly compromised upon chemical conjugation to a DNA architecture. In previous studies,^14^ it has been also shown that the fluorescence of BAC was quenched by nearly 90% upon conjugation to proteins, despite the introduction of long spacers.^57^ It is also known that the chloride sensitivity of quinolinium-based chloride indicators decreases upon conjugation to dextran due to limited accessibility of quencher to the fluorophore.^58^

We observed that covalently linked BAC to DNA results in dramatic quenching leading to reduced chloride sensitivity of BAC. We did not observe any significant effect of linker flexibility on the chloride sensitivity of BAC. Interestingly, the extent of BAC quenching increased in the case of scaffolds that carried an internal BAC label. However the chloride sensitivity of BAC remained unaffected and this was regardless of the nature of flanking base pairs. Upon conjugation to PNA, a major population of BAC was in a similar environment as that of free BAC resulting in significant improvement in chloride sensitivity. Importantly, no bound/intercalated form of BAC was observed in ssPNA, dsPNA or DNA–PNA hybrids unlike as seen in dsDNA based constructs. Currently, we cannot deconvolute the relative contributions of PET-based quenching of BAC by guanine residues and the shielding effect due to the negatively charged DNA backbone. The difference in sequences of DNA and PNA strands used in this study support that these effects are sequence independent.

To achieve intracellular targeting and reasonable chloride sensitivity we designed *Clensor* where BAC is covalently linked at the N-terminus of a DNA–PNA hybrid duplex. It showed significant improvement in fluorescence intensity as well as chloride sensitivity compared to DNA based scaffolds. The BAC–PNA module serves as the sensing module while DNA serves as the targeting module. The basic principles involved in the design are generalizable to a variety of ionic reporters and can be used for developing any collisional quenching based nucleic acid sensor.

We therefore describe some general guidelines while designing ionic reporters using DNA. To sense ions by collisional quenching, one may increase bimolecular collisional quenching constants by choosing a sensing dye oppositely charged compared to the charge of the ion being detected.^26^ We may achieve this by modifying functional groups on the dye such that it decreases electrostatic repulsion between the ion of interest and reporter dye.^19^ For example, if one is sensing negatively charged ions, carboxylate groups could be modified to esters or amides. Dyes that fluoresce at longer wavelengths are preferred due to lower phototoxicity and autofluorescence contributions.^59^ Both sensing and normalizing dyes should be spectrally well separated and placed spatially at least 10 nm apart to avoid cross-talk.^60^ Further substantial spatial separation between sensing and normalizing dyes would enable better binding between sensor and its protein targets as well as preserve the photophysical properties of both dyes. The use of an electrically neutral PNA backbone disrupts intercalation between the dye and carrier scaffold due to lesser breathing motions associated with PNA–DNA hybrids,^50^ which can be leveraged to achieve better sensing. DNA–PNA hybrids have much lower interstrand electrostatic repulsions that results in higher *T*_m_ values of duplex domains. This can therefore reduce the molecular weight of nucleic acid-based reporter, which is highly desirable when investigating living systems.^61^ Given that guanines tend to quench fluorophores due to PET,^41^ one should take care that sequence designs avoid dyes being placed proximal to guanines. Sequence designs should further avoid 5′ terminal guanines or GR (where *R* = G/A) as this reduces guanine ionization potentials by 0.2–0.5 eV due to stacking.^62^ Higher ionization potential of G reduces the probability of PET. Use of rigid linkers and incorporation of bulges are useful strategies to disrupt stacking interactions between dyes and backbone. However it is important to note that coplanar stacking of dyes with purines (G > A) tends to increase static quenching.^41^

## Supplementary Material

Supplementary information
