# Supplementary material for: Rational design of a quantitative, pH-insensitive, nucleic acid based fluorescent chloride reporter
Source: Chem Sci. 2015 Dec 1;7(3):1946–53. doi: 10.1039/c5sc04002g (PMC6042475; doi:10.1039/c5sc04002g)
Supplement: Supplementary file 1 [file SC-007-C5SC04002G-s001.pdf]

Electronic Supplementary Material (ESI) for Chemical Science.
This journal is © The Royal Society of Chemistry 2015

Electronic Supplementary Information for

Rational design of a quantitative, pH-insensitive, nucleic acid based chloride reporter

Ved Prakash†, Sonali Saha†, Kasturi Chakraborty and Yamuna Krishnan*

1. Experimental section

Reagents. All unmodified oligonucleotides (Table S2) were purchased from Sigma (India). All modified oligonucleotides (Table S2) were obtained from IBA GmbH (Germany). Fluorescently labeled oligonucleotides were subjected to ethanol precipitation prior to use to remove any contaminants from synthesis. Fluorescently labeled oligonucleotides were subjected to ethanol precipitation prior to use to remove any contaminating fluorophores. All oligonucleotides quantified by their UV absorbance at 260 nm. The peptide nucleic acids (PNA) oligomers, P and P' (Table S2) were synthesized using standard solid phase Fmoc chemistry on Nova Syn® TGA resin (Novabiochem, Germany) using analytical grade reagents (Applied Biosystems®, USA), purified by reverse phase HPLC (Shimadzu, Japan). HPLC purified and lyophilized oligonucleotides were dissolved in MQ water, aliquoted into small fractions and stored at -20 °C. To quantify BAC conjugated DNA and PNA oligomers, molar extinction coefficient at 260 nm (ε_{260}) for BAC was considered as 1.65×10^5 M^{-1} cm^{-1}.

BAC synthesis and characterization. 10,10′-Bis[3-carboxypropyl]-9,9′-biacridinium dinitrate (BAC) and 10,10′-Bis[(3-N-succinimidyloxycarbonyl)propyl]-9,9′-biacridinium dinitrate (BAC NHS) was synthesized according to a literature procedure [1,2].

Step I: Acridone (Figure S1, compound I) (5 g, 0.0256 mol), potassium hydroxide (1.8 g, 0.032 mol) and absolute ethanol (80 mL) were heated to reflux (80 °C) for 30-40 min until the acridone dissolved. The ethanol and water formed were distilled off and the remaining solid was lyophilized overnight. 100 mL (large excess) γ-butyrolactone was added to the dried material and the solution was heated to reflux (205°C) for 4 h. The excess γ-butyrolactone was distilled off under reduced pressure. The remaining solid was dissolved in water, precipitated by dropwise addition of dilute HCl, filtered, and lyophilized. The lyophilized product was further purified by recrystallization from chlorobenzene to yield (70%) 10-[3-carboxypropyl]-9(10H)-acridone (Figure S1, compound II). Compound II was characterized using mass spectrometry and NMR. HRMS m/z calculated for $C_{17}H_{15}NO_3$ 282.1130 ($[M+H]^+$), observed 282.1118. ^{1}H NMR (400 MHz, DMSO-*d6*): δ 8.35(d, 2H, aromatic, J = 8.0 Hz), 7.33 (dd, 2H, aromatic, $J_1 = J_2 = 4.0$ Hz), 7.83 (t, 2H, aromatic, $J_1 = J_2 = 8.0$ Hz), 7.94 (d, 2H, aromatic, J = 8.0 Hz), 4.48 (t, 2H, N-CH_2, J = 8.0 Hz), 2.02 (m, 2H, alkyl CH_2), 2.57 (t, 2H, alkyl CH_2, J = 8.0 Hz), 12.28 (s, 1H, COOH).

Step II: To a suspension of compound II (1.4 g, 5 mmol) in acetone (40 ml), zinc dust (6.6 g, 101 gram atom) was added. The mixture was then stirred at 30–40 °C for 20 min. The flask was cooled in ice-cold water, and 37% HCl (60.5 g, 613 mmol) was added dropwise over 6 h under N_2 atmosphere at ~10 °C. The reaction mixture was stirred overnight at room temperature followed by addition of 25 ml of degassed water. A bright yellow precipitate was collected by filtration, rinsed with water, and dissolved in 25 ml of 5% aqueous NaOH. The mixture then was filtered, and the filtrate was neutralized with acetic acid till bright yellow precipitate generation. The precipitate was filtered, washed with water, dried, and recrystallized from hot ethanol to yield 2.2 g (65%) of 10,10′-bis[3-carboxypropyl]-9,9′-biacridylidene (Figure S1, compound III) as yellow crystalline solid. Compound III was characterized using mass spectrometry. HRMS m/z calculated for $C_{34}H_{30}N_2O_4$ 266.1181 ($[M+2H]^{2+}$), observed 265.6102.

Step III: Compound III (2.6 g, 5 mmol) was heated in 2 N nitric acid (120 ml) for 2 h at 120 °C until most of the brownish mass was dissolved. After cooling and filtration, the precipitate was washed with dilute nitric acid, dried, and recrystallized from dilute nitric acid to yield 2.4 g (80%) of 10, 10′-bis[3-carboxypropyl]-9,9′-biacridinium dinitrate (Figure S1, compound IV) as a yellow crystalline solid. Compound IV was characterized using mass spectrometry and NMR. HRMS m/z calculated for $C_{34}H_{30}N_2O_4$ 265.1103, observed 265.0545. ^{1}H NMR (400 MHz, DMSO-*d6*): δ 9.08 (d, 4H, aromatic, J = 8.0 Hz), 8.48 (t, 4H, aromatic, J = 8.0 Hz), 7.75 (t, 4H, aromatic, J = 8.0 Hz), 7.68 (m, 4H, aromatic), 5.61 (t, 4H, N-CH_2, J = 8.0 Hz), 2.07 (t, 4H, alkyl CH_2, J = 4.0 Hz), 2.89 (t, 4H, alkyl CH_2, J = 8.0 Hz), 12.47 (s, 2H, COOH).

Step IV: To prepare 10, 10′-Bis[(3-N-succinimidyloxycarbonyl)propyl]-9,9′-biacridinium dinitrate (Figure S1, compound V), compound IV (1.0 mg, 1.53 μmol) was dissolved in DMF (500 μl) and triethylamine (12.5 μl). After addition of *N*-hydroxysuccinimide (NHS) (0.7 mg, 4.0 equiv.), and 1-Ethyl-3-(3-dimethylaminopropyl) carbodiimide (EDC) (0.58 mg, 2.0 equiv.); the mixture was stirred in the dark for 18 h followed by spin down at 10,000 rpm for 10 min to remove the precipitated if any. The supernatant (DMF solution of BAC-NHS) was used directly to conjugate to DNA and PNA .Compound V was characterized using mass spectrometry. HRMS m/z calculated for $C_{42}H_{36}N_4O_8^{2+}$ 362.1266, observed 362.1573.

BAC-DNA conjugates preparation and purification. Amine labeled DNA (2.5 nmol) was dissolved in 180 μL of 100 mM sodium phosphate buffer, pH 8.5. 20 μL (25 nmol) of freshly prepared above mentioned DMF solution of BAC-NHS ester was added to DNA and stirred for 4 h at room temperature. The reaction mixture was then subjected to reverse phase HPLC (Shimadzu, Japan) purification to isolate the BAC-DNA conjugates. A linear H_2O/CH_3CN gradient starting from 5/95 (v/v) to 100/0 (v/v) over 30 min was employed. In a typical procedure, 100 μL of crude reaction mixture was injected into an analytical C18 column (Phenomenex, LunaC18, 100 Å pore size, 5 μm bead size with dimensions of 250 mm × 4.6 mm) and separated using the above mentioned gradient with a flow rate of 2 mL/min. In order to avoid multiple conformation adopted by single BAC-DNA conjugate during purification, the HPLC column was kept inside a temperature controllable unit (CTO 20A,

Shimadzu) to maintain a constant temperature of 40 °C throughout the procedure. Fractions corresponding to BAC-DNA conjugates starting from 20.0 min to 21.0 min were collected, concentrated and stored at -20 °C.

PNA synthesis and characterization. PNA synthesis was performed using standard solid phase Fmoc chemistry on Nova Syn® TGA resin [3,4] using analytical grade reagents. In brief, Fmoc-Lys (Boc)-NovaSyn® TGA (cat. No. 04-12-2662) was weighed out in a dry vac-elute column containing a rice grain magnetic stir bar. The resin was swelled in dry DCM overnight (6-8 h) with gentle stirring. DCM was removed by applying vacuum and the resin was washed with dry DCM thrice. Freshly prepared 600 µL 20 % piperidine in dry DMF was added to the resin and stirred gently for 30 min. The piperidine solution was then replaced with fresh solution and was stirred for another 30 min. The piperidine solution was removed by applying vacuum, and the resin was alternatively washed thrice with dry DCM, twice with dry DMF and finally with dry DCM. To this deprotected resin, 600 µL of coupling solution containing PNA monomer (Fmoc-N-A/G/C-Bhoc-COOH or Fmoc-N-T-COOH) along with HATU, HOAT and DIPEA-lutidine mix in NMP was added. The coupling mixture used was added five equivalents in excess of the loading value of the resin for efficient coupling. The coupling reaction was carried out for 2 h with gentle stirring, with an exception to the first coupling which was carried out for 6 h. Each coupling step was followed by three DCM, two DMF and one DCM wash in the same order. To the resin, 600 µL of 20% piperidine in DMF was added and this cycle was repeated until the complete sequence was synthesized. After coupling of the lysine residue (Fmoc-Lys(Mtt)-OH) at the N-terminus, 600 µL of a freshly prepared mixture of TFA/ TIS/ DCM (1:5:94) was added and stirred gently for 5 min to remove methytrityl (Mtt) group, for conjugation of BAC at the e-N site . This step was repeated thrice with fresh solutions for complete deprotection. After that, the resin was washed as mentioned earlier. The resin was neutralized by addition of 10% DIPEA in DCM. To the Mtt deprotected resin, BAC-NHS ester in DMF (10 eqv.) was added and stirred gently for 16-18 h. The final Fmoc deprotection was carried out as described earlier and the BAC conjugated PNA sequence (BAC-PNA) was cleaved from the resin bed by treatment with a mixture of TFA/TIS/water (95:2.5:2.5) with vigorous stirring for 1.5 h. The step was repeated twice and the flow through solution obtained was collected in a round bottom flask. The cleaved resin was washed twice with water, 500 µL EtOH and 500 µL DCM for 30 min each and the washings were collected in the same flask. The collected solution was evaporated completely in a Rotavapor® (Buchi, Switzerland) Synthesized BAC-PNA was precipitated by cold and dry ether. The precipitated material was dried and dissolved in MQ water after careful decanting of the supernatant. This crude reaction mixture was then subjected to purification by RP-HPLC (Shimadzu, Japan). CH_3CN/H_2O gradient starting from 5/95 (v/v) to 45/55 (v/v) over 5 min and 45/55 (v/v) to 100/0 (v/v) over 25 min was employed. 0.1% TFA was added to CH_3CN/H_2O mixture to sharpen the eluted peaks. In a typical experiment, 100 µL of crude product solution was injected into an analytical C18 column (Phenomenex, Luna C18, 100 Å pore size, 5 µm bead size with dimensions of 250 mm × 4.6 mm) and separated using the above mentioned gradient with a flow rate of 2 mL/min. In order to minimize multiple conformations formed by BAC-ssPNA, the column was kept inside a temperature controllable unit (CTO 20A, Shimadzu) to maintain a constant temperature of 40 °C throughout the procedure. Fractions starting

from 16.5 min to 17.5 min were collected and subjected to positive ion ESI-MS analysis. BAC-ssPNA was stored as a 2 mM aqueous solution at -20 °C.

Mass spectrometry. Mass spectrometry studies for BAC were performed on a Micromass ESI-MS Q-TOF Ultima Mass Spectrometer (Manchester, UK) with micro-channel plate detector in positive ion mode. Samples were diluted into a final concentration of ~400 μM using 1:1 mixture of methanol and water containing 0.1% formic acid. Synthesized ssPNA and BAC-ssPNA purified by reverse phase HPLC were characterized using positive ion mode ESI-MS on coupled Synapt G2 HD mass spectrometer (Waters). The ssPNA and BAC-ssPNA samples were diluted to a final concentration of ~20 μM using a solution of a 1:1 mixture of water and acetonitrile containing 0.1% formic acid. The mass spectra were acquired with a source temperature of 70 °C, capillary voltage at 1.5 kV and cone voltage at 60 V. MassLynx 4.0 software was used for the analysis of the spectra obtained. The HPLC fraction containing the right mass was taken further for subsequent studies. The HPLC profile and mass spectrum of the crude product after cleavage indicated that the synthesized BAC-ssPNA was quite pure.

NMR studies. All NMR spectra were recorded on Bruker 400 MHz NMR Spectrometer.

Sample preparation. Stock solutions of all nucleic acid constructs were prepared at 10 μM concentration by mixing all the relevant component strands (Table S2) in equimolar ratio in 10 mM sodium phosphate buffer, pH 7.2. Annealing was done by heating the solution at 90 °C for 5 min and cooling at the rate of 5 °C/ 15 min. All the samples were incubated at 4 °C minimum for 48 h before experiments.

Fluorescence studies. All fluorescence studies were carried out using a Fluoromax-4 (Horiba Scientific, Japan) spectrophotometer. 10 μM stock of different nucleic acid constructs were diluted to a final concentration of 200 nM using 10 mM sodium phosphate buffer, pH 7.2 and incubated for 30 min at room temperature prior to experiments. 200 nM free Tetramethylrhodamine (TMR) was added as normalizing fluorophore to the diluted samples in absence of Alexa 647 on the complementary strands. The emission spectra of BAC and TMR or Alexa 647 were acquired by exciting the samples at 435 nm (λ_{Ex}^{BAC}) and 540 nm (λ_{Ex}^{TMR}) or 640 nm ($\lambda_{Ex}^{Alexa\ 647}$) respectively. An emission value of 10 mM sodium phosphate buffer, pH 7.2 served as blank and was subtracted from all relevant acquired spectra. To study the chloride sensitivity of different constructs, different final [Cl^-] ranging between 5 mM to 200 mM were achieved by adding microliter aliquots from a 1M stock of NaCl to 400μL of sample. Emission intensity of BAC at 505 nm (G) was normalized to emission intensity of TMR at 570 nm (R) or Alexa 647 at 670 nm (R). Fold change was calculated from the ratio of R/G values at two different added [Cl^-].

Lifetime decay measurements. The fluorescence lifetime decay traces were acquired using TEMPRO (Horiba Jobin Yvon, Japan) with a Time Correlated Single Photon Counting (TCSPC) system. The excitation source used was a pulsed NanoLED with excitation at 443 nm at a repetition rate of 1 MHz. The emission was collected at 488 nm, and was monitored at the magic angle (54.7°) to eliminate the contribution from the decay of anisotropy. The fluorescence decay data collection was done using microchannel plate photomultiplier tube coupled to a TCSPC card at 0.11 ns per channel. The instrument response function (IRF)

at 443 nm was measured using a dilute colloidal suspension of coffee whitener, and was found to be 1.23 ns (FWHM).The peak preset was set to 20,000 counts.

Fluorescence decay measurements on various constructs were carried at 1 μM concentration in 10 mM degassed potassium phosphate buffer, pH 7.2. For each fluorescence lifetime decay based titration, 2 μL aliquots of 1 M NaCl were sequentially added in batches to 100 μL of sample at 25°C.

Data analysis. Fluorescence decay curves were analyzed with the associated software using a standard iterative reconvolution method, assuming a multiexponential decay function:

$$I(t) = A + \sum_{i=1}^{n} \left(B_i e^{-t/\tau i} \right) \qquad i = 1 - 3$$

where, A is the background level (dark count of the detector), B_i is the fractional amplitude, τ_i is the fluorescence lifetime of the ith decay component such that $\Sigma B_i = 1$. The quality of fit was judged on the basis of the reduced chi-square statistic (χ^2_{red}), the randomness of residuals and autocorrelation function about the mean[5]. Mean lifetime was calculated using the equation[6]:

$$< \tau > \ = \frac{\sum_{i=1}^{n} \left(B_i \tau_i^2 \right)}{\sum_{i=1}^{n} \left(B_i \tau_i \right)} \qquad i = 1 - 3$$

The Stern-Volmer constant was estimated from slope of the linear plot of τ_0/τ versus [Q],

$$\frac{< \tau_0 >}{< \tau >} = 1 + K_{SV}[Q]$$

where $<\tau_0>$ is the mean fluorescence lifetime in the absence of quencher and$<\tau>$ is the mean fluorescence lifetime in the presence of quencher at a concentration [Q]. Bimolecular quenching rate constant (k_q) is given by the equation

$$k_q = \frac{K_{SV}}{< \tau_0 >}$$

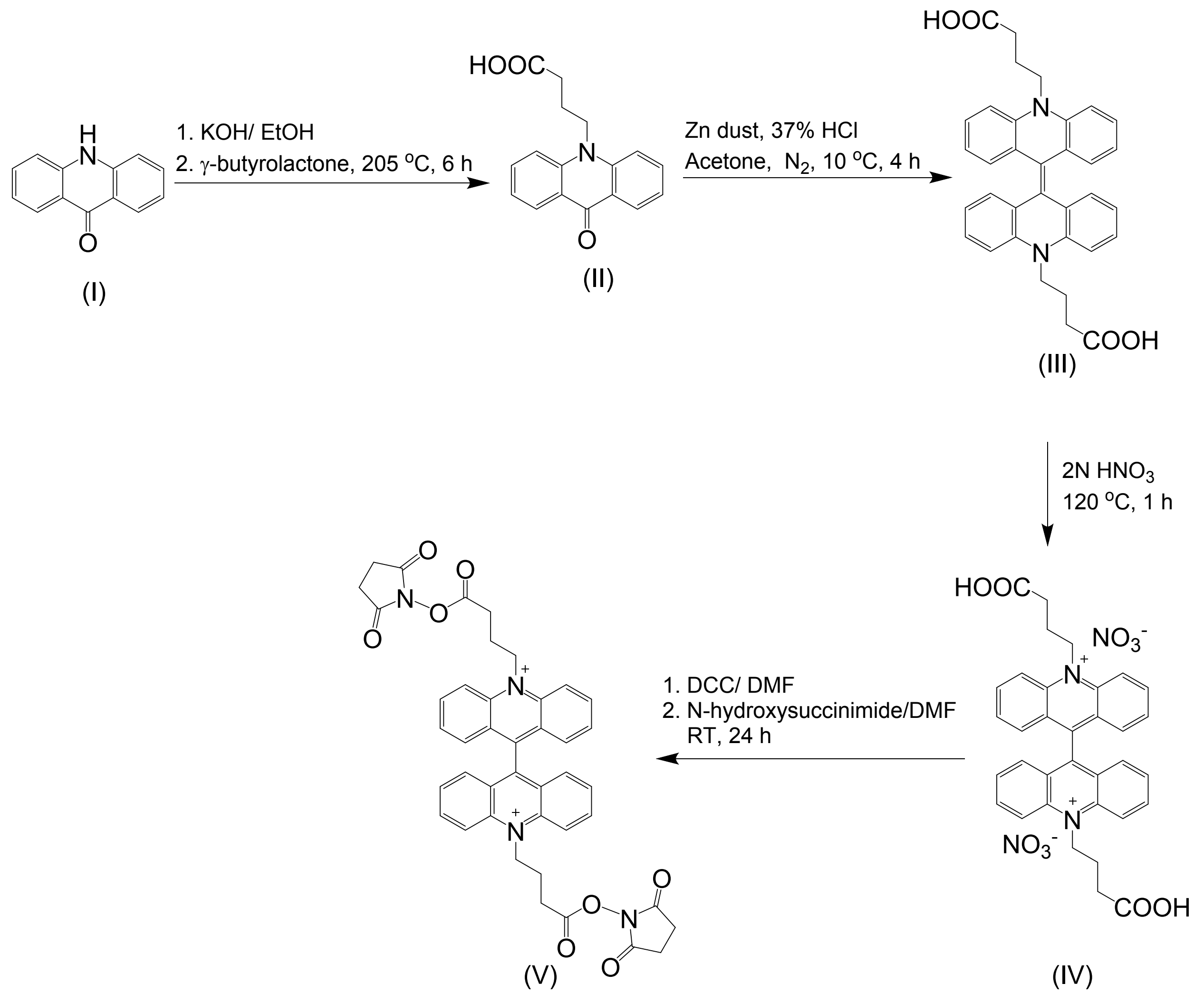

Figure S1 Reaction scheme for synthesis of BAC NHS ester

Name	Sequence	Observed m/z (ion peak)	Observed mass (Da)	Calculated mass (Da)
P	NH_2-Lys-ATC AAC ACTGCA-Lys-COOH	696.7471 ($[M'+5H]^{5+}$)	3478.7355	3478.4915
		580.7830 ($[M'+6H]^{6+}$)	3478.6980	3478.4915
		497.9610 ($[M'+7H]^{7+}$)	3478.7270	3478.4915
BAC-ssPNA	BAC-NH_{ε}-Lys-ATC AAC ACTGCA-Lys-COOH	679.8029 ($[M+H+2Na+K]^{6+}$)	3992.7318	3992.0861
		582.8340 ($[M+2H+2Na+K]^{7+}$)	3992.7445	3992.0861
		510.1084 ($[M+3H+2Na+K]^{8+}$)	3992.7658	3992.0861
		453.5418 ($[M+4H+2Na+K]^{9+}$)	3992.7669	3992.0861
P′	Cys-TGCAGTGTTGAT-Lys	710.3474 ($[M''+5H]^{5+}$)	3546.737	3546.73
		887.6876 ($[M''+4H]^{4+}$)	3546.75	3546.73
		1183.2268($[M''+3H]^{3+}$)	3546.68	3546.73

Table S1 Molecular weight of ssPNA and BAC-ssPNA as determined by electrospray ionization mass spectrometry (ESI-MS). M and M′ are the exact mass of ssPNA and BAC-ssPNA respectively.

Name	Sequences	Description
$C1_3$	5′ GACTCACTGTTTGTCTGTCGTTCTAGGATATATATTT 3′	3′ amine-C_6 linker
C2	5′ AAATATATATCCTAGAACGACAGACAAACAG*T*GAGTC 3′	Unmodified
$C1_I$	5′ GACTCACTGTTTGTCTGTCGTTC***T***CGGATATATAT 3′	Internal 5- aminoallyl modification on the T shown in bold and italics
$C2_{B0}$	5′ ATATATATCCGAGAACGACAGACAAACAG*T*GAGTC 3′	Unmodified
$C2_{B1}$	5′ ATATATATCCGGAACGACAGACAAACAG*T*GAGTC 3′	Unmodified
$C2_{B2}$	5′ ATATATATCCGAACGACAGACAAACAG*T*GAGTC 3′	Unmodified
$C2_{B3}$	5′ ATATATATCCAACGACAGACAAACAG*T*GAGTC 3′	Unmodified
P	BAC-$NH_ε$-Lys-ATC AAC ACT GCA-Lys-COOH	Sensing module: PNA strand
P′	NH_2-Lys-TGC AGT GTT GAT-Lys-COOH	Complementary PNA strand
D2	5′ TATA***T***ATA GGATCTTGCTGTCTGGTG TGC AGT GTT GAT 3′	Normalizing module: internal Alexa 647 modification on the T shown in bold and italics
D1	5′ CACCAGACAGCAAGATCC TATATATA 3′	Targeting module: Unmodified

Table S2: Sequences of PNA and DNA oligomers used in this study. The sequences in same colors are complementary. The bases shown in blue are the bulge sequences.

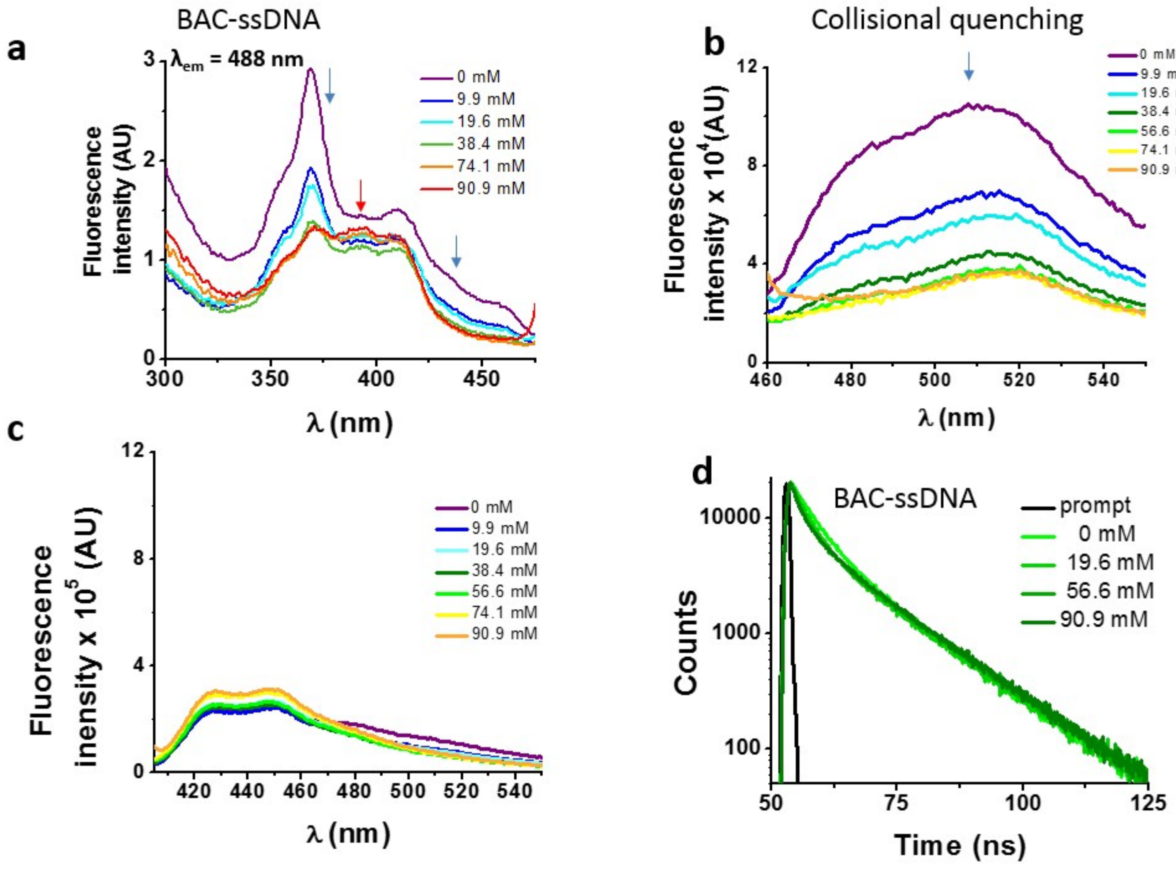

Figure S2 Photophysical properties of BAC in BAC-ssDNA. (**a**) Excitation spectra of 1 μM BAC-ssDNA ($C1_I$) at different chloride concentrations. Emission spectra at (**b**) λ_{ex} = 443 nm and (**c**) λ_{ex} = 392 nm. (**d**) Representative fluorescence lifetime decay traces of 1 μM BAC-ssDNA in 10 mM potassium phosphate buffer, pH 7.2 at various chloride concentrations (λ_{ex} = 392 nm, λ_{em} = 488 nm).

Effect of DNA on BAC upon conjugation

BAC when covalently linked to DNA as in BAC-ssDNA shows two populations with distinct lifetimes. See Table S3, where a mono-exponential function is insufficient to describe the observed decay trace. One of these populations is chloride sensitive (Fig S2b) while other is chloride insensitive (Fig S2c). The chloride sensitive population has excitation maxima at 370 nm and 443 nm (blue arrows, Fig S2b) while the insensitive population has an excitation maximum at 392 nm (red arrow, Fig. S2b). Both these forms emit fluorescence at 488 nm. Fluorescence lifetime decay traces at 488 nm while exciting the sample at 392 nm, indicates that the major population in BAC-ssDNA is insensitive to chloride (Fig S2d).

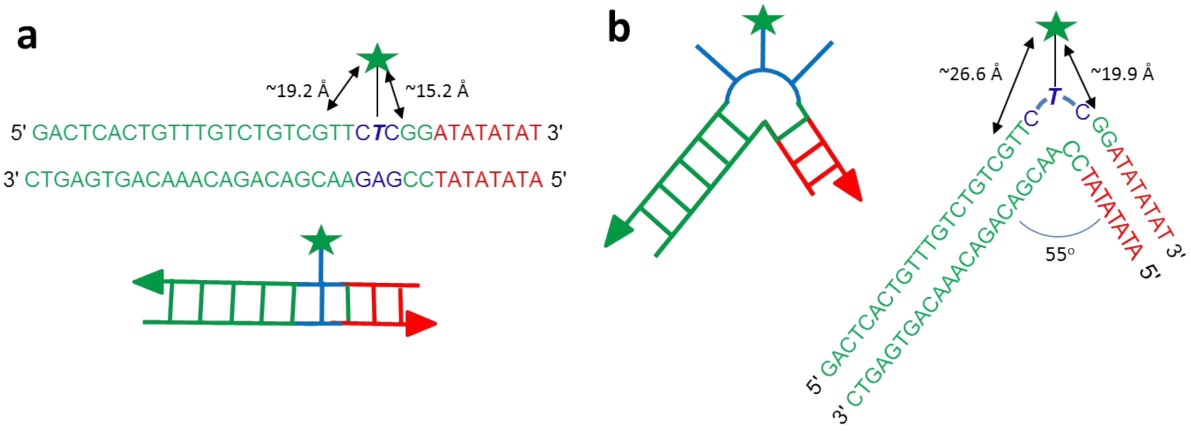

Figure S3: Schematic showing structures and calculated distances between BAC and guanosine residues in different nucleic acid assemblies used in this study. (**a**) BAC-dsDNA$_{B0}$ (**b**) BAC-dsDNA$_{B3}$. We have the aminoallyl linker to be rigid, in the all-trans form and positioning BAC in the plane of the paper, bisecting the angle made by helix axes of the constituent helical domains.

Compared to BAC-dsDNA$_{B0}$, BAC is expected to be located relatively farther away from guanosine residue in BAC-dsDNA$_{B3}$. In BAC-dsDNA$_{B3}$ we observe two chloride sensitive populations one of which has a longer lifetime (7.8 ns) and is a minor population (11%) and the other, which has a major population (85%) with a shorter lifetime (1.3 ns) (Table 1). Together they show a non-linear chloride dependence of average lifetime as a function of chloride concentration. In BAC-dsDNA$_{B0}$ the abundance of these two populations are comparable, with identical short lifetime species, but where the long lifetime species shows a lifetime of 5.8 ns. Thus we assign the short lifetime species to a probable conformation where the duplex regions flanking the bulge are frayed, resulting in closer approach of the guanosines that now quench with greater efficiency due to greater proximity, that is now independent of the angle introduced by the B0 or B3 bulge. When the helical domains are intact, this appropriately positions guanosines away from the BAC label that results in the distinct lifetimes of 7.8 ns and 5.8 ns depending on the relative distance introduced by the angle between the helix axes.

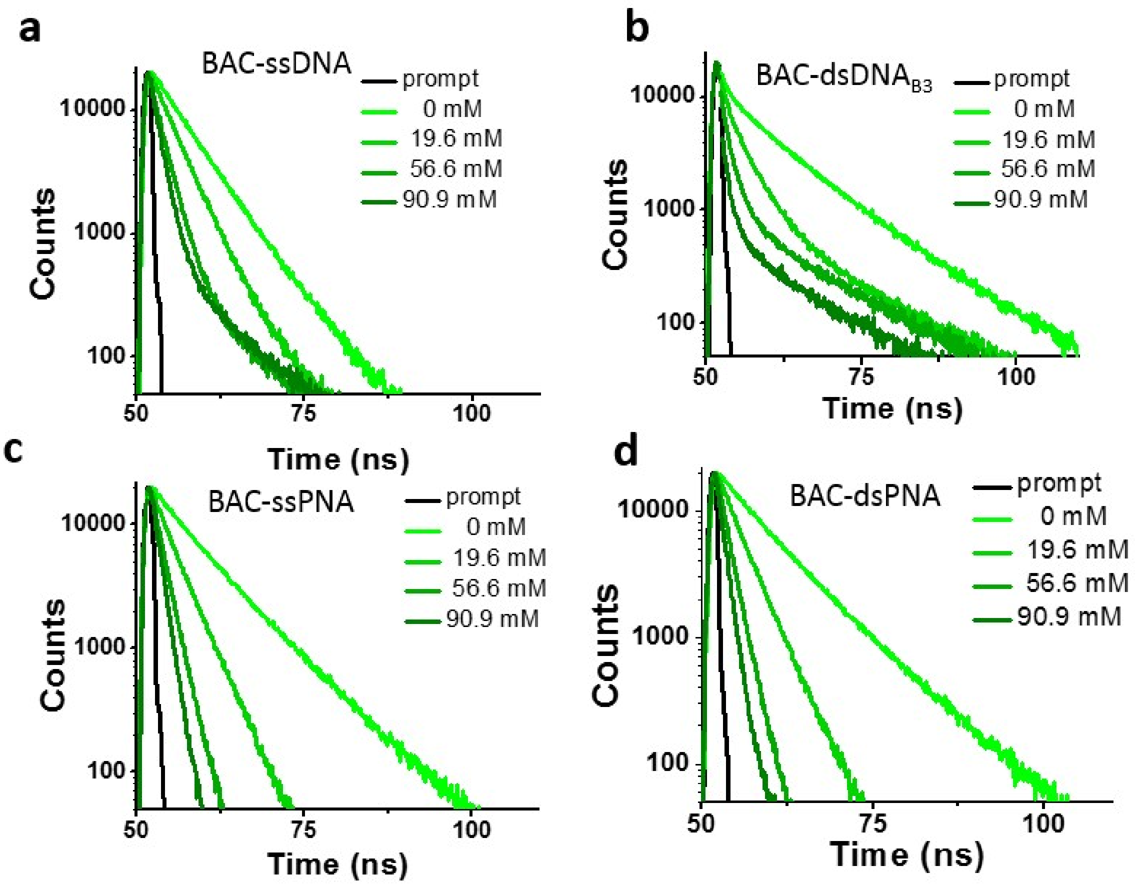

Figure S4 Representative fluorescence lifetime decay traces of 1 µM (**a**) BAC-ssDNA, (**b**) BAC-dsDNA$_{B3}$, (**c**) BAC-ssPNA and (**d**) BAC-dsPNA in 10 mM potassium phosphate buffer, pH 7.2 given chloride concentrations (λ_{ex} = 443 nm, λ_{em} = 488 nm) .

BAC when conjugated to DNA as in BAC-ssDNA, shows a chloride insensitive component with a lifetime of 13.9 ns (Fig S4a and b) that corresponds to a population that interacts with the DNA backbone. When BAC is conjugated to charge neutral PNA as seen in BAC-ssPNA, this population that interacts with nucleotide backbone is abolished, yielding instead a population with increased fluorescence lifetime (7.35 ns) and chloride sensitivity (Fig S4c and d).

Table S3 Comparison of the residuals (green) and autocorrelation (blue) obtained for the fits to the experimental fluorescence lifetime decay traces of free BAC, BAC-ssDNA, BAC-dsDNA$_{B0}$ (at 90.9 mM chloride) and BAC-ssPNA. All residuals correspond to 0 mM chloride unless otherwise mentioned.

Sample	1 exp	2 exp	3 exp
**BAC**	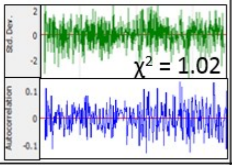		
**BAC-ssDNA**	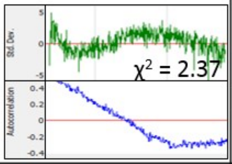	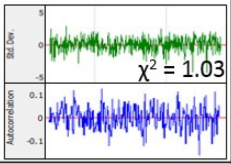	
**BAC-dsDNA**$_{B0}$	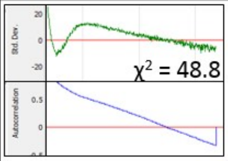	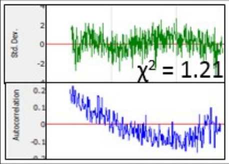	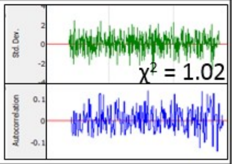
**BAC-ssPNA**	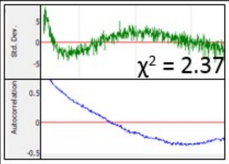	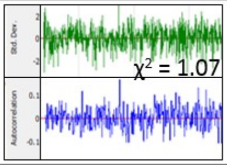	

Free BAC shows a mono-exponential fluorescence lifetime decay while BAC-ssDNA shows a biexponential decay (Table 1 and table S3). BAC-dsDNA shows complex multiexponential fluorescence decay trace indicating existence of multiple populations of BAC due to strong interaction with DNA backbone. BAC-ssPNA shows a biexponential decay trace due to reduced interactions between BAC and nucleotide backbone (Table 1 and table S3). The autocorrelation traces indicate that BAC-ssDNA and BAC-ssPNA can only be explained by a bi-exponential fit; BAC-dsDNA$_{B0}$ can only be explained adequately by invoking a third component (Table 1 and table S3). However BAC alone is adequately described by a simple mono-exponential decay (Table 1 and table S3).

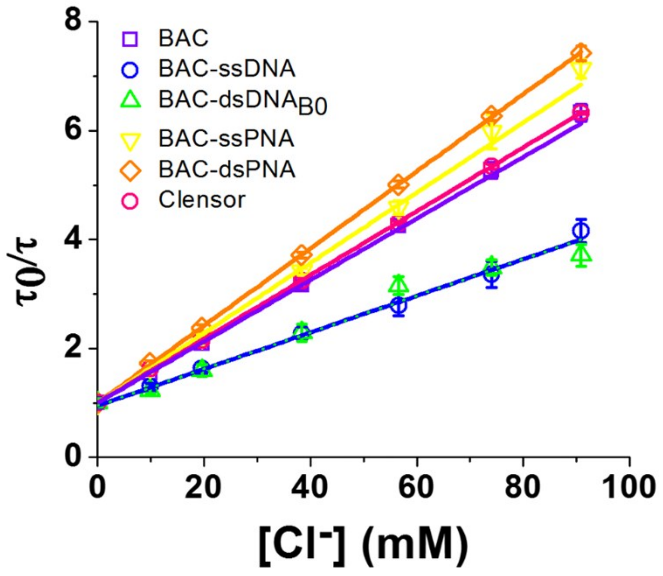

Figure S6 Comparative Stern-Volmer plots for all the constructs used in this study. Error bars indicate the mean of three independent experiments ± s.e.m.

In general, DNA constructs show poor sensitivity to chloride due to reduced lifetime and lower k_q. PNA constructs show enhanced sensitivity to chloride due to increased k_q (Table1).

References:

1 M. H. Litt, J. Kim and J. M. Rodriguezparada, *J. Polym. Sci. Polym. Chem. Ed.*, 1985, **23**, 1307–1319.

2 N. D. Sonawane, J. R. Thiagarajah and a S. Verkman, *J. Biol. Chem.*, 2002, **277**, 5506–13.

3 L. Christensen, R. Fitzpatrick, B. Gildea, K. H. Petersen, H. F. Hansen, T. Koch, M. Egholm, O. Buchardt, P. E. Nielsen, J. Coull and R. H. Berg, *J. Pept. Sci. an Off. Publ. Eur. Pept. Soc.*, 1995, **1**, 175–183.

4 T. Koch, H. F. Hansen, P. Andersen, T. Larsen, H. G. Batz, K. Otteson and H. Orum, *J. Pept. Res. Off. J. Am. Pept. Soc.*, 1997, **49**, 80–88.

5 J. R. Lakowicz, *Principles of Fluorescence Spectroscopy*, Springer, Third edit., 2006.

6 A. Sillen and Y. Engelborghs, 1998, **67**, 475–486.
